# Developing Integrated Care: The I-CONNECT Care Model for Older Adults with Mood Disorders and Multimorbidity

**DOI:** 10.5334/ijic.10212

**Published:** 2026-11-12

**Authors:** Louise de Almeida Ferreira Fonseca, Laura Tops, Mieke Vermandere, Bert Vaes, Mathieu Vandenbulcke, Jos Tournoy, Johan Flamaing, Geert Goderis, Mieke Deschodt

**Affiliations:** 1 KU Leuven, Department of Public Health and Primary Care, Academic Center for General Practice, Leuven, Belgium; 2 KU Leuven, University Psychiatric Center, Division of Psychiatry, Geriatric Psychiatry, Leuven, Belgium; 3 KU Leuven, Leuven Brain Institute, Department of Neurosciences, Research Group Psychiatry, Neuropsychiatry, Leuven, Belgium; 4 KU Leuven, Department of Public Health and Primary Care, Division of Geriatric Medicine, Leuven, Belgium; 5 Competence Center of Nursing, University Hospital Leuven, Leuven, Belgium

**Keywords:** integrated healthcare, chronic conditions, depression in older adults, patient-centered care, care coordination, primary healthcare

## Abstract

**Introduction:**

Older adults living at home increasingly face mood disorders alongside multimorbidity, yet healthcare remains fragmented. Gaps in coordination, limited integration of mental and physical health services, and insufficient multidisciplinary collaboration persist. To address these challenges, we developed I-CONNECT, a structured, stakeholder-informed, integrated care model. The aim of this paper is to describe the systematic development of I-CONNECT and its alignment with evidence-based practices and feasibility within the Belgian healthcare system.

**Methods:**

We followed a pragmatic, mixed-methods approach guided by ten recommended actions for intervention development. The process involved a scoping review, qualitative research with healthcare providers, patients, and caregivers, and a series of stakeholder workshops and meetings to iteratively design and refine the intervention and inform its feasibility. A logic model articulated the program theory, and the intervention was described using the TIDieR framework.

**Results:**

The process resulted in I-CONNECT, a prototype integrated care model for home-dwelling older adults with mood disorders and multimorbidity. The model builds on existing healthcare structures and on six key principles: personalized care, care coordination, self-management support, multidisciplinary collaboration, medication reviews, and follow-ups. These were translated into a six-step care process.

**Conclusion:**

I-CONNECT is a pragmatic, evidence-informed model addressing systemic fragmentation in Belgian healthcare. A forthcoming evaluation will refine the model and inform broader integrated care initiatives.

## Introduction

The global population is undergoing a rapid demographic shift, with projections indicating that the number of individuals aged 65 years and older will more than double by 2054, reaching 1.7 billion [1]. Among this population of older adults, mood disorders, particularly depression, are highly prevalent but often undertreated [2]. Aging is also strongly associated with multimorbidity, commonly defined as the presence of two or more chronic conditions [3]. Over 60% of adults aged 65 and older live with multimorbidity, and this burden is even higher among those with mood disorders [4,5]. The bidirectional relationship between mood disorders and multimorbidity is well established [5,6]. Approximately 25% of individuals with bipolar disorder experience multiple physical health conditions, while 14% have additional psychiatric comorbidities [5]. Moreover, individuals with severe mental illness have a 1.84 times higher risk of developing multiple physical conditions compared to those without mental illness [7], and those with depression are two to three times more likely to experience physical multimorbidity [6].

The coexistence of mood disorders and physical multimorbidity negatively impacts quality of life, increases healthcare needs [8,9], and is associated with higher mortality rates [10] and increased healthcare costs [11]. Despite the well-documented interplay between aging, mental health disorders, and chronic physical conditions, current healthcare models remain fragmented, even in countries with strong primary care foundations, such as Belgium. Although Belgium has a well-developed primary care network, gaps in care coordination, limited integration between mental and physical health services, and challenges in multidisciplinary collaboration persist [12]. These barriers hinder effective communication among providers [13], contribute to inefficient resource utilization [14] and lead to suboptimal patient outcomes [15]. Older adults with both mental and physical health conditions face limited access to mental health services and additional challenges, including stigma, inconsistent implementation of evidence-based guidelines, and restricted access to multidisciplinary care models [14]. The combination of mental and physical conditions further complicates care delivery, as needs often extend across multiple providers, and care settings that are not routinely aligned [16].

To overcome these challenges, integrated care has emerged as a critical approach to enhancing continuity, coordination, and accessibility of services across multiple providers and care settings [17]. This approach shifts away from disease-centered, hospital-centric models toward proactive, holistic, and person-centered care [18]. International frameworks such as the World Health Organization Integrated Care for Older People (ICOPE) [19] approach and the SELFIE framework [20] provide structured guidance for the organization of care for individuals with complex health needs. While ICOPE focuses on maintaining functional ability in older adults, the SELFIE framework specifically addresses multimorbidity, emphasizing comprehensive assessment, multidisciplinary collaboration, coordination across services, and support for self-management. However, evidence shows that integrated care models remain highly heterogeneous in their definitions, components, and implementation, with limited clarity on which elements drive effectiveness [21].

A growing body of evidence supports its effectiveness in managing chronic diseases, particularly among high-risk populations [22]. However, the real-world impact of integrated care depends on how well it aligns with the specific context in which it is implemented [23]. Factors such as healthcare system structure, resource availability, provider engagement, and patient needs influence its feasibility and effectiveness [24]. Understanding these contextual elements is essential when designing interventions that are both clinically effective and adaptable to real-world settings.

In this context, the present study aims to describe the systematic stakeholder-informed development of I-CONNECT (*I*ntegrated *C*are M*o*del for Home-Dwelli*n*g older Patie*n*ts with D*e*pression and Physi*c*al Multimorbidi*t*y), an integrated care intervention tailored to the needs of older adults living at home with mood disorders and multimorbidity in Leuven, Belgium. The objective is to develop a context-specific model that bridges the gap between current fragmented care practices and the complex realities faced by this population, while ensuring alignment with person-centered and evidence-based principles. A subsequent evaluation will assess the intervention effectiveness while also examining implementation process in real-world settings.

## Methods

This article focuses on the intervention development phase, integrating findings from previously published empirical studies together with new data generated through stakeholder engagement and participatory development activities.

### Overall methodological framework

The development of I-CONNECT followed the structural recommendations of O’Cathain et al. 2019, who propose ten key actions for developing complex health interventions [25]. Rather than applying these actions as a strictly linear sequence, we used them as a structured framework to organize, document, and critically reflect on the intervention development process.

I-CONNECT was developed through a pragmatic multi-method approach, integrating findings from a scoping review, focus groups with healthcare providers, semi-structured interviews with patients and informal caregivers, stakeholder workshops, and targeted stakeholder consultation meetings. The development process followed an iterative, stakeholder-informed approach, where evidence and stakeholder input were progressively integrated to refine the intervention.

Findings from the different data sources were integrated through an interpretative and iterative synthesis process led by the core research team. The synthesis followed principles of thematic and interpretative analysis, using constant comparison across data sources to identify patterns and higher-order categories. This process resulted in the identification and structuring of key intervention elements, including guiding principles and intervention steps. Methodological rigor was enhanced through triangulation of multiple data sources, iterative comparison across sources, and structured consensus procedures within a multidisciplinary research team.

Figure 1 provides an overview of the intervention development process, illustrating the sequence of activities, data sources, and stakeholder involvement over time.

**Figure 1:**
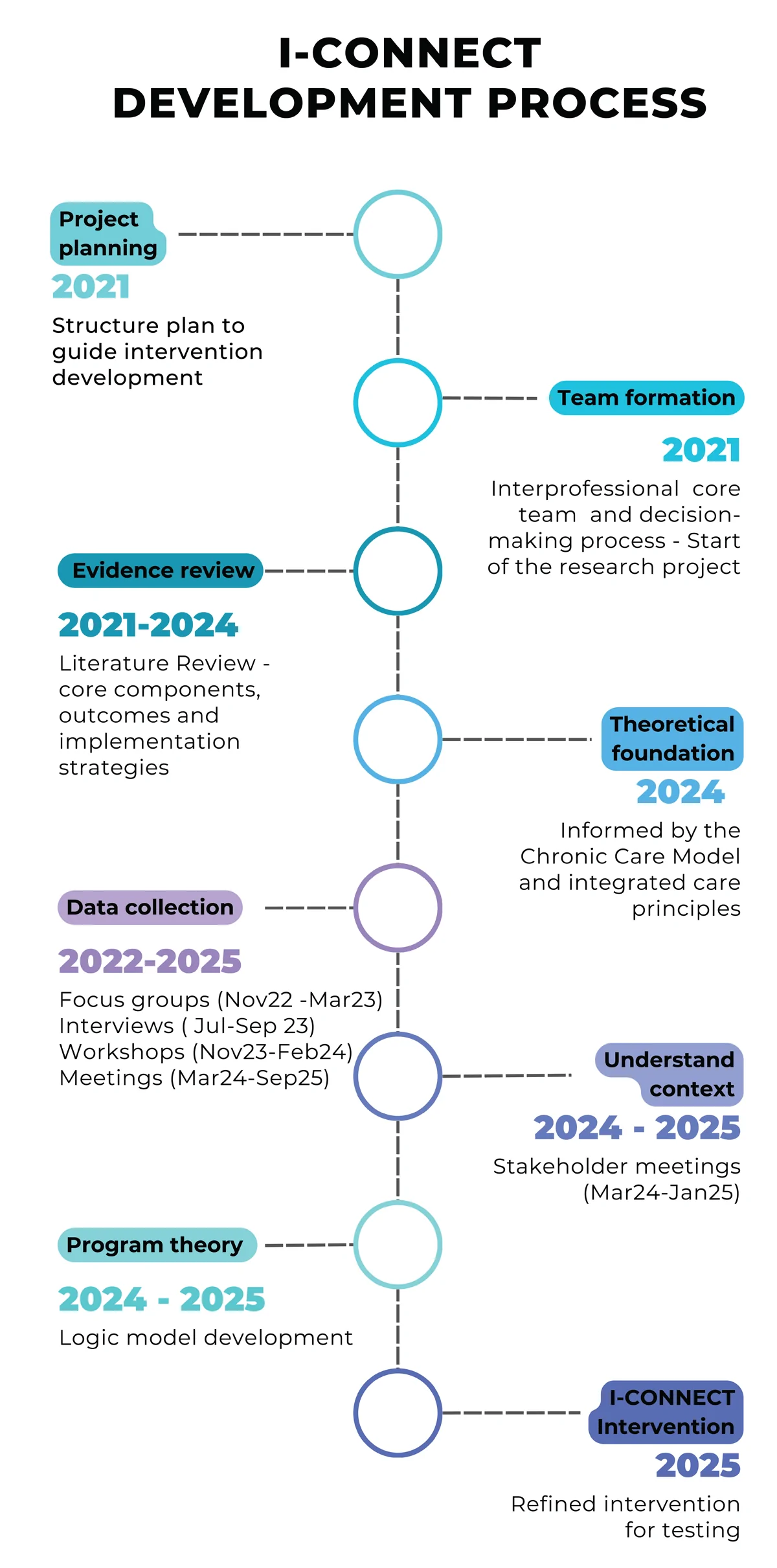
I-CONNECT care model development process.

#### Plan the development process

We outlined a structured plan to guide intervention development and evaluation. The development process was planned step by step, including a timeline and the sequencing of activities, and specifying how each phase would be operationalized.

Data collection methods were predefined, starting with a literature review to inform the initial understanding of the problem, followed by qualitative data collection (interviews, focus groups, and stakeholder workshops), each building on the findings of the previous phase.

For each step, protocols were developed in advance and, where required, submitted for ethical approval. This ensured that data collection and stakeholder engagement were conducted in a structured and consistent manner across the different phases of the development process.

#### Build a team and decision-making structure

An interprofessional core team (n = 8) was established, including general practitioners (with expertise in chronic care and primary care data systems), a nurse researcher with expertise in health services and implementation science, a psychiatrist, and two geriatricians. A pharmacist contributed at a specific stage of the development process, particularly in relation to medication management. A statistician was involved in later stages to support methodological considerations related to evaluation and study design.

This multidisciplinary composition ensured that intervention decisions were informed by complementary clinical, methodological, and implementation perspectives. The team met regularly in structured steering meetings to review evidence, integrate stakeholder input, and make decisions regarding intervention content, delivery, and feasibility.

Rigor was supported through triangulation of perspectives, explicit discussion of alternative options, and documented decision-making processes. Final decisions were made within the core team to ensure coherence across development stages.

#### Review existing research evidence

Existing research evidence was used to inform the conceptual and practical design of I-CONNECT. First, we conducted a scoping review to identify core components of integrated care models for older adults with depression and multimorbidity, and to map reported outcomes and implementation strategies [26]. Second, an ongoing systematic review examines implementation strategies used in integrate care interventions (PROSPERO registration: CRD42024569242). In the present study, these evidence sources served to identify relevant care components, inform initial assumptions about feasible intervention elements, and highlight gaps requiring contextual adaptation. Combining published evidence with primary qualitative data supported a context-sensitive development process.

#### Draw on existing theories

The intervention was informed by the Chronic Care Model, which emphasizes productive interactions between informed patients and proactive healthcare teams to improve outcomes in chronic illness management [27]. Development was further aligned with international principles of integrated care [17], highlighting person-centeredness, continuity, multidisciplinary collaboration, and coordination across levels of care. These frameworks provided a conceptual foundation to guide the selection, organization, and interpretation of intervention components throughout the development process.

#### Undertake primary data collection

Primary data were collected using multiple qualitative methods to inform intervention development.

Three separate focus groups were organized with 17 healthcare providers (November, Dezember 2022 and March 2023) including general practitioners, nurses, psychologists, social workers, pharmacists, geriatricians and geriatric psychiatrist from the target region, i.e. Leuven in Belgium. We explored current care practices, needs and challenges in caring for older adults with depression and physical multimorbidity. The methodology and findings are reported in a separate publication [28].

Semi-structured interviews with home-dwelling older adults diagnosed with major depression and physical multimorbidity, as well as their informal caregivers (July-September 2023) provided insights into lived experiences, unmet needs, and priorities within the healthcare system. The data from these interviews will be further synthesized and reported in a separate publication.

Three stakeholder workshops were held between November 2023 and February 2024 at University Psychiatric Center (UPC) KU Leuven. A total of 10 stakeholders participated, including general practitioners, psychiatrists (including trainees), geriatricians, nurses, physiotherapists, a social worker, a policy representative, and a participant with lived experience in mental health.

The workshops had a participatory function and focused on: (a) mapping the current patient journey, (b) developing the first draft of the intervention workflow, (c) defining the roles and responsibilities of different actors within the intervention, and (d) identify barriers and facilitators to implementation.

These workshops represent original data generated within the present study and were central to translating empirical findings into practical intervention components and refining the structure of the I-CONNECT model.

#### Involving stakeholders and understanding context

Stakeholder involvement was central throughout the development process to ensure that the intervention was clinically relevant, contextually appropriate, and acceptable to those delivering and receiving care.

In addition to workshops, targeted stakeholder meetings were conducted with regional care networks (e.g., Zorgzaam Leuven, Waasland Integrated Care), patient organizations, and policy representatives. These consultations provided insights into existing care structures, ongoing initiatives, and system-level constraints, including reimbursement mechanisms and digital infrastructure.

Preliminary versions of the intervention were presented during these consultations to assess feasibility and alignment with current practice, and feedback was used to refine the model.

#### Articulate program theory

The program theory was developed by the core research team and iteratively refined based on stakeholder input to clarify the intervention’s underlying mechanisms and ensure contextual relevance. A logic model was used to map how inputs and activities were expected to lead to outputs and outcomes [29]. The pipeline model provided the initial structure, while iterative refinement allowed the model to evolve as insights from the development process accumulated.

#### Data integration and synthesis process

The integration of data sources followed a structured, team-based interpretative process. First, findings from each data source (scoping review, focus groups, interviews, and stakeholder activities) were summarized independently. Second, these findings were compared across sources to identify recurring patterns and relevant insights for intervention development.

Third, an initial set of intervention components and actions was generated and translated into a first draft of the intervention during stakeholder workshops. This preliminary model was subsequently discussed within the core research team during structured meetings. Alternative options were explicitly considered, and decisions were guided by clinical relevance, feasibility within the Belgian healthcare system, and alignment with theoretical frameworks and existing evidence.

This process was iterative, with successive refinement of intervention components based on ongoing stakeholder feedback, leading to the final structure of the I-CONNECT model. The evolving intervention model was iteratively refined and validated through stakeholder engagement, ensuring face validity and alignment with real-world clinical practice.

#### Reporting and transparency

In order to enhance transparency and replicability, the intervention was described following the Template for Intervention Description and Replication (TIDieR) guidelines [30]. A detailed description of the intervention following the TIDieR checklist is provided in [Supplementary File I – TIDieR-Checklist]. Supplementary table 1 summarizes stakeholder engagement and data collection activities.

### Ethical Considerations

Ethical approval for the development of the I-CONNECT intervention was obtained from the Ethical Committee of UZ/UPC/KU Leuven, Belgium (Registration: S66783/B3222022000935, EC Ref: EC:2022-679). The Committee approved the study protocol in September 2022, with additional documents approved in May 2023. The research was conducted in accordance with Belgian regulations on biomedical research and international ethical guidelines.

## Results

This section presents the results of the integrative synthesis process, in which findings from the scoping review, qualitative studies, and stakeholder engagement activities were combined, structured, and translated into a coherent intervention model.

### Determining the target population and practical implementation considerations

The target population for I-CONNECT was initially defined as home-dwelling older adults (65 years or older) with major depression and physical multimorbidity. The physical multimorbidity was defined as the co-occurrence of two or more chronic physical conditions, consistent with international consensus definitions [3]. During stakeholder meetings, this scope was broadened to include older adults with clinically diagnosed mood disorders – including major depression, persistent depressive disorder, depression related to a medical condition or substance use, bipolar I or II disorder, bipolar disorder related to a medical condition or substance use, or cyclothymia – based on DSM-5 criteria [31], as well as documented physical multimorbidity. This refinement reflected the broader clinical spectrum encountered in practice and allowed for a more inclusive and patient-centered approach while preserving the focus on complex care needs in community settings.

Patients described difficulties navigating fragmented services, particularly when mental and physical care were poorly coordinated, as identified in patient and informal caregiver interviews and reinforced during the stakeholders meetings. Healthcare providers further emphasized that introducing additional, stand-alone structures would risk worsening fragmentation given existing workload pressures. Multidisciplinary approaches were common but often lacked genuine alignment between mental and physical healthcare. These insights were derived from focus groups with healthcare providers and further reinforced during stakeholder workshops and meetings conducted.

Building on these findings, stakeholder workshops and consultations highlighted the importance of integrating the intervention within existing healthcare pathways rather than creating parallel systems. Additional barriers were financial sustainability and lack of interoperable digital records, as well as facilitators including stakeholder engagement, growing interest in patient-centered approaches, and opportunities to link with ongoing healthcare reforms and pilot digital tools (e.g., Alivia system [32]). Consequently, rather than developing a separate platform or care pathway, I-CONNECT leverages current infrastructures and appoints a dedicated care coordinator to streamline communication between primary, specialized, and community pharmacy services.

### The I-CONNECT logic model and its guiding principles

The six guiding principles did not emerge from a single data source, but from the integration of findings across the scoping review, qualitative interviews, focus groups, and stakeholder workshops.

The I-CONNECT logic model (Figure 2) provides a structured representation of the program theory underlying an integrated care approach for older adults living at home with mood disorders and multimorbidity. The model was developed iteratively by combining evidence from the literature with findings from the qualitative studies and stakeholder engagement. Initial intervention prototypes were generated during stakeholder workshops and subsequently refined and aligned within the core research team, informed by the Chronic Care Model and integrated care principles.

**Figure 2:**
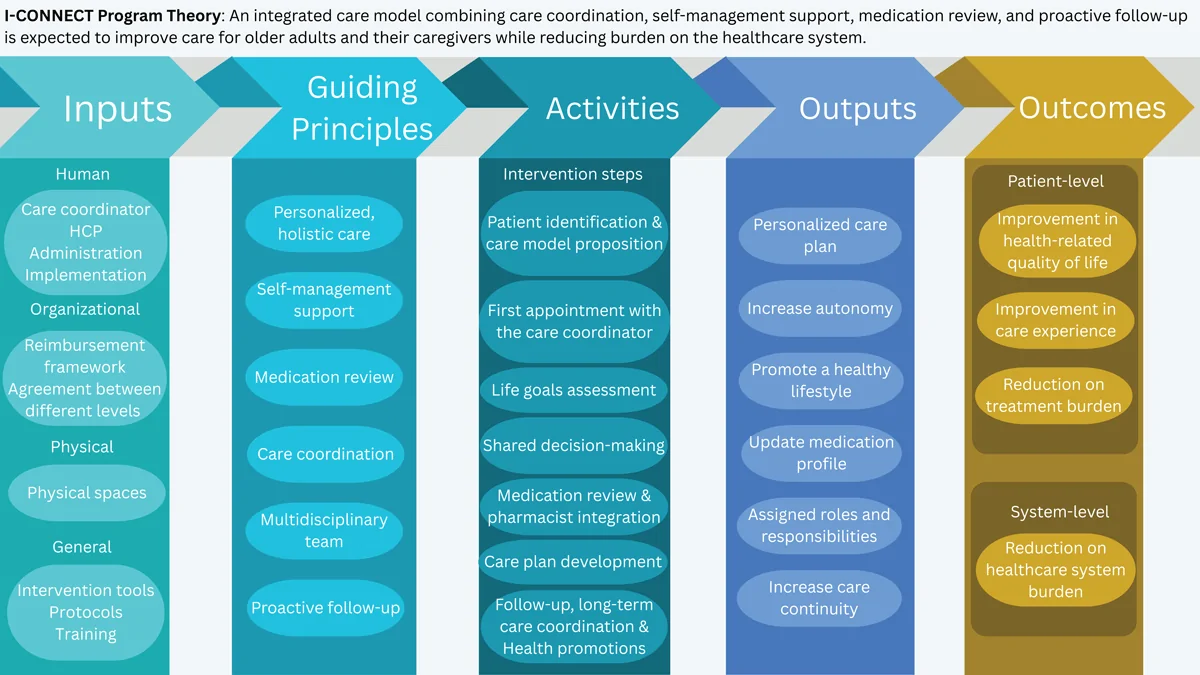
I-CONNECT Logic Model representing the program theory in a pipeline model.

This process resulted in the identification and structuring of key intervention components and guiding principles. Inputs include the human, organizational, physical, and general resources needed to deliver integrated care—such as healthcare providers (psychiatrists, primary care physicians, pharmacists, geriatricians), a dedicated care coordinator, existing reimbursement frameworks and agreements between different levels of care, physical spaces for service delivery, and general tools or protocols for assessment, training, and education. At the core of the model are six guiding principles that shape its central activities and ensure an integrated, patient-centered approach (Table 1).

1. *Personalized*, *Holistic Care* was consistently emphasized by patients and caregivers, who reported feelings of exclusion from decision-making and dissatisfaction with fragmented services. The scoping review corroborated these concerns by revealing the lack of fully integrated mental and physical healthcare.
2. *Self-Management Support* was driven by patient accounts of needing more involvement in the treatment plan and autonomy, and by workshop recommendations calling for the consistent use of shared decision-making tools in routine consultations.
3. *Medication Review* emerged as critical in both systematic reviews on multimorbidity interventions, which identified medication management as a key component, and in stakeholder input confirming that community pharmacists could lead these reviews under Belgium’s existing reimbursement framework.
4. The *Care Coordinator* role was explicitly endorsed by healthcare providers in focus groups, which identified inadequate case management as a major obstacle, and was further refined through workshops that clarified responsibilities for communication and follow-up.
5. *Multidisciplinary Teams* were advocated by all stakeholder groups—particularly during workshops mapping the patient journey—to address role ambiguity and improve communication among primary care, mental healthcare, and community services.
6. *Proactive Follow-Up* was underscored in patient interviews, focus groups, and workshops as crucial for preventing relapse or gaps in care, while allowing timely adjustments to treatment plans as patient needs evolve.

**Table 1:** I-CONNECT Guiding Principles and Supporting Evidence.


GUIDING PRINCIPLE	DESCRIPTION	SUPPORTING EVIDENCE

Personalized, Holistic Care	Individualized care plans based on life goals and preferences, beyond disease focus	Patient and informal care givers interviews; scoping review; workshop feedback

Self-Management Support	Shared decision-making tools and strategies autonomy.	Patient and informal care givers interviews; workshop; focus groups

Medication Review	Pharmacist-led reviews to optimize polypharmacy, aligned with reimbursement rules	Systematic reviews; stakeholder meetings

Care Coordinator	Dedicated role ensuring continuity, communication, and case management.	Focus groups; patient and informal care givers interviews; workshops

Multidisciplinary Teams	Collaboration across GPs, psychiatrists, pharmacists, and others for unified care	Stakeholder workshops; scoping review; focus groups

Proactive Follow-Up	Flexible, ongoing support to prevent relapse and adapt to evolving needs	Patient and informal care givers interviews; focus groups; workshops


These six guiding principles were translated into practice through a structured six-step care model (Figure 3 & Box 1): patient identification and proposition of the care model, the initial appointment with the care coordinator, life goals assessment and shared decision-making, medication review and pharmacist integration, personalized care plan development, multidisciplinary collaboration and follow-up through long-term care coordination and health promotion. The program will take 12 months, with flexible in-person, telephone, or virtual contacts, provided either at the specialized center (UZ Leuven) or via home visits.

**Figure 3:**
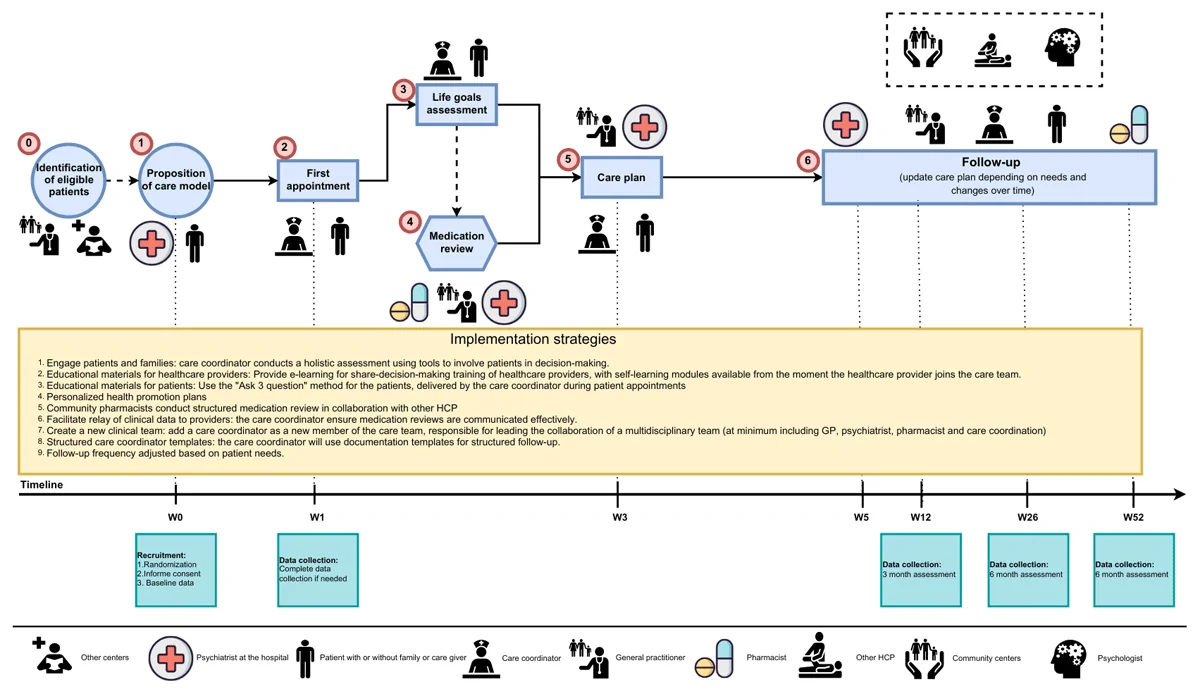
*Intervention Overview by steps*. The image provides an overview of the intervention, including the key actors involved, and implementation strategies. The figure presents the six-step I-CONNECT care process, highlighting both core intervention components (e.g., care coordination, medication review) and embedded implementation strategies, including patient engagement, provider training, multidisciplinary integration, and flexible follow-up. Training resources for coordinators, pharmacists, and GPs are available via the project website (gbiomed.kuleuven.be/iconnect).

**Box 1 The SIX-STEP Approach Of The I-CONNECT Intervention**

**Step-1: Patient Identification and Proposition of Care Model**

Patients with mood disorders and at least two chronic physical conditions are identified and referred through existing pathways (e.g., GPs, geriatricians). Only psychiatrists finalize eligibility.

**Step 2: First Appointment with the Care Coordinator**

The dedicated care coordinator introduces patients to I-CONNECT’s structure, confirms eligibility, and gathers comprehensive health and social information. The Positive Health – a framework that views health as the ability to adapt and self-manage across physical, emotional, and social dimensions, focusing on what matters most to the individual [33] is introduced to capture each patient’s unique goals. Relevant healthcare providers receive an e-learning module on shared decision-making to reinforce *Self-Management Support*.

**Step 3: Life Goals Assessment and Shared Decision-Making**

Using the Positive Health framework, patients identify priorities across physical, mental, and social well-being. These discussions further *Personalized, Holistic Care* and *Self-Management Support*, equipping patients with the “Ask 3 Questions” [34] method to enhance engagement in treatment planning.

**Step 4: Medication Review and Pharmacist Integration**

Community pharmacists, working within Belgium’s reimbursement framework, conduct structured reviews for patients on five or more chronic medications or those exceeding 160 defined daily doses (DDDs) annually (detailed info www.apotheia.be/ggg-medicatienazicht). Aligning with the *Medication Review* and *Multidisciplinary* principle, pharmacists collaborate with GPs to optimize prescribing, and patients are consulted before changes are made.

**Step 5: Personalized Care Plan Development**

All data gathered—life goals, self-management capabilities, and medication insights—culminate in an individualized plan that addresses medical, psychosocial, and caregiver involvement. This step again underscores *Multidisciplinary Teams* and *Personalized, Holistic Care*, ensuring the plan remains dynamic and revisable over time.

**Step 6: Follow-Up and Long-Term Care Coordination & Health Promotion**

Sustained follow-up, guided by the principle of *Proactive Follow-Up*, is central to the I-CONNECT model. The care coordinator manages flexible follow-up—initially biweekly, then adapted to patient needs—via in-person, virtual, or phone contacts. Regular case discussions with the multidisciplinary team ensure timely adjustments and reinforce *Multidisciplinary Collaboration*. These discussions are organized flexibly depending on patient complexity and care needs, and may include structured phone consultations, shared updates via secure digital communication, or care summary letters shared between providers. The care coordinator plays a central role in initiating and coordinating these exchanges.

This process also supports *Self-Management Support* by promoting health behaviors aligned with the patient’s care goals. Patients remain in the program for 12 months, with continuous coordination ensuring engagement across primary and specialized care.

The intervention is designed to generate several outputs that enhance care delivery, informed by the synthesis of findings from qualitative data (interviews and focus groups), stakeholder inputs, and literature reviews. Care plans become personalized to individual life goals and evolving needs, while self-management support and shared decision-making strengthen patient autonomy. Medication management is optimized through regularly updated medication profiles led by community pharmacists, roles and responsibilities among healthcare professionals are clearly delineated, and continuity of care is improved, thereby mitigating fragmentation and promoting long-term engagement. Over time, these outputs are intended to yield meaningful outcomes at the patient level—such as improved health-related quality of life, reductions in treatment burden, and enhanced care experiences—and at the system level, including lower rates of avoidable hospital admissions and emergency department visits. Ultimately, the I-CONNECT logic model reflects the hypothesis that an integrated framework bridging mental and physical healthcare will lead to more efficient resource use and a more sustainable healthcare delivery system compared to standard care.

## Discussion

This study describes the systematic development of I-CONNECT, an integrated care model for older adults with mood disorders and multimorbidity. Drawing on evidence, qualitative data, and stakeholder input, the study focused on how these sources were translated into concrete design decisions shaping the intervention. The resulting model reflects both identified care needs and the constraints and opportunities of the Belgian healthcare context. Stakeholder involvement throughout the process ensured that these decisions were grounded in clinical priorities and routine care practice.

A key design decision was to embed the intervention within existing healthcare structures rather than developing parallel services. This was consistently supported across stakeholder discussions and qualitative data, where participants emphasized that additional stand-alone programs would increase fragmentation and provider burden. This concern aligns with previous findings highlighting the risks of fragmented and duplicative services [35,36]. By building on existing referral pathways, reimbursement frameworks, and digital infrastructures, I-CONNECT was designed to be implementable within current practice, thereby supporting feasibility and potential adoption. This approach is consistent with implementation science principles emphasizing early consideration of context and stakeholder involvement to enhance feasibility and sustainability [37].

Another key design decision was to position a dedicated care coordinator at the center of the model. This emerged directly from the development process, where stakeholders consistently identified inadequate case management as a barrier, and patients reported difficulties navigating fragmented services. Introducing a clearly defined coordination role addresses these challenges by making the care coordinator responsible for communication, follow-up, and care alignment across providers. This decision was further supported by existing evidence showing that care coordination improves continuity and reduces emergency care use in multimorbid populations [38,39,40].

Multidisciplinary collaboration was similarly identified as necessary but difficult to operationalize in practice. Stakeholders highlighted role ambiguity and limited coordination between primary care, mental health services, and community providers. In response, I-CONNECT translates this need into a structured component of the model by clarifying responsibilities among general practitioners, psychiatrists, pharmacists, and care coordinator. This is particularly relevant in populations with multimorbidity and psychiatric conditions, where fragmented communication and competing care priorities are common [41].

Self-management support was operationalized through shared decision-making. This choice was informed by stakeholder input indicating that traditional self-management approaches can be burdensome for older adults with complex conditions. In this context, self-management was not framed as an individual responsibility, but as a supported and collaborative process integrated into routine care. This approach is particularly relevant in populations with multimorbidity, where disease-specific education is difficult to implement due to overlapping conditions and competing treatment priorities. Embedding structured tools such as “Ask 3 Questions” within consultations provides a practical way to facilitate this approach, by supporting patients in expressing preferences and engaging in care decisions without increasing cognitive or treatment burden. Furthermore, this is consistent with recent calls to view shared decision-making as a self-management strategy [42] and aligns with evidence in depression care that goal-setting facilitates recovery and engagement [43].

Personalized and holistic care was operationalized through a structured assessment based on the Positive Health framework. This decision emerged during stakeholder discussions following the initial draft of the intervention, where participants emphasized that care planning should be guided by what matters most to the patient, rather than solely by clinical priorities. In response, the model incorporates a structured exploration of patient goals across physical, mental, and social domains, which forms the basis for individualized care planning. This approach supports alignment between patient priorities and clinical decision-making, particularly in the context of multimorbidity, where competing conditions and treatments make disease-specific approaches insufficient [44]. Pharmacist-led medication review was included as a structured component of the model, reflecting both stakeholder input and evidence identifying polypharmacy as a central challenge in this population. Rather than introducing a new process, this component builds on the existing reimbursed “home pharmacist” framework in Belgium, supporting feasibility and sustainability within routine care. For this component to function effectively, access to relevant clinical information and collaboration between pharmacists, general practitioners, and geriatric psychiatrists are essential. Embedding medication review within this multidisciplinary context allows prescribing decisions to be aligned with overall care planning. This is consistent with evidence showing that medication review is most effective when embedded within multicomponent interventions [45].

Finally, we included a proactive and flexible follow-up as a guiding principle of the model. This was informed by stakeholders input and qualitative findings highlighting the fluctuating nature of both depression and multimorbidity, and the risk of patients disengaging from care over time. To address this, I-CONNECT incorporates flexible follow-up and re-engagement strategies, including in-person, telephone, and home-based contacts, as well as referral to additional psychosocial support when needed. In addition, the model integrates elements of geriatric assessment, allowing changes in patients’ functional, mental, or social status to be identified over time and incorporated into the care plan. This enables care to be adapted dynamically, rather than relying on fixed or episodic interventions, and supports continuity across care settings. This approach is consistent with evidence linking multimorbidity-related stressors to depressive symptoms and care vulnerability [46], as well as with recommendations for proactive monitoring in older populations [47].

A subsequent evaluation will assess the effectiveness and implementation of this approach in real-world settings, focusing on patient health-related quality of life, perceived quality of care, treatment burden, and healthcare utilization.

### Strengths and limitations

A key strength of I-CONNECT lies in its systematic and stakeholder-informed development process. Stakeholder engagement was embedded throughout the development process, from defining the scope to refining the intervention prototype, and was complemented by evidence from a scoping review, qualitative research, and iterative stakeholder engagement activities. Such participatory approaches can enhance feasibility, contextual fit, and legitimacy of complex interventions [48], whereas top-down models often fail to adapt to local realities [49]. This multi-source approach strengthens confidence that the intervention is relevant to routine care and grounded in the perspectives of those involved in delivering and receiving care. In addition, the explicit articulation of a program theory provides a clear foundation for future evaluation.

On the other hand, feasibility assessments were based on stakeholder input and contextual analysis prior to the actual implementation. As such unforeseen barriers may emerge once the care model is tested in real-world practice. Also, the model was developed in the Belgian context, which may limit transferability to health systems with different financing or organizational structures. The transferability of I-CONNECT is therefore more likely to lie in its guiding principles and development logic than in its exact organizational form.

## Conclusion

Grounded in a systematic development process that integrated evidence, theory, and stakeholder input, I-CONNECT represents a stakeholder-informed care model for older adults with mood disorders and multimorbidity. By embedding care coordination, shared decision-making, pharmacist-led medication reviews, multidisciplinary collaboration, and proactive follow-up within existing Belgian healthcare structures, the intervention prioritizes feasibility and addresses critical gaps in current practice. Future evaluation will assess its effectiveness and refine its implementation strategies, offering insights for broader integrated care initiatives in aging populations.

## Additional Files

The additional files for this article can be found as follows:

10.5334/ijic.10212.s1 Supplementary File I. TIDieR–Checklist.

10.5334/ijic.10212.s2 Supplementary Table 1. Summary of Stakeholder Engagement and Data Collection Activities.
