# Supplementary material for: Developing Integrated Care: The I-CONNECT Care Model for Older Adults with Mood Disorders and Multimorbidity
Source: Int J Integr Care. 2026 Nov 12;26(4):10. doi: 10.5334/ijic.10212 (PMC13761781; doi:10.5334/ijic.10212)
Supplement: Supplementary file 2 [file ijic-26-4-10212-s2.pdf]

Supplement Material:

Table 1. Summary of Stakeholder Engagement and Data Collection Activities

Data Source	Participants Involved	Date	Mode of Data Collection	Purpose of Data Collection
Focus Groups	17 healthcare providers (HCPs) from primary and residential care	Nov 2022 – Mar 2023	In-person and online semi-structured discussions	Understand HCPs' needs, expectations, and experiences
Patient Interviews	10 older adults with depression & multimorbidity, and 7 informal caregivers	Jul – Sep 2023	In-person, semi-structured interviews	Identify patient and caregiver needs and expectations
Stakeholder Workshops	10 stakeholders: psychiatrists, GPs, physiotherapists, nurses, social workers, policymakers	Nov 2023 – Feb 2024	In-person workshops	Refine the intervention, refine roles, and assess feasibility
LOK Meeting	Local GPs	Mar 2024	In-person meeting	Obtain feedback on the intervention model
Meeting with Tool Developers and System Support	NexusHealth team, Alivia & Research team	Jun 2024	Online meeting	Assess feasibility of integrating existing communication systems between care settings
Meetings with Vlaams Patient Platform	Patient representatives & research team	Jul 2024	Online meeting	Discuss patient-centered implementation strategies
Stakeholder Meetings	Experts & research team	Jul 2024	Online meeting	Refine target population and intervention components, and assess expected impact
Stakeholder Meeting – ZorgZaam Leuven	Program coordinators & research team	Aug 2024	Online meeting	Discuss integration of the intervention into primary care
Stakeholder Meeting – Belgian Pharmaceutical Association	Program coordinators & research team	Aug 2024	Online meeting	Discuss medication review and its integration into primary care
Stakeholder Meeting – Waasland Integrated Care Coordinator	Policymakers & integrated care representatives	Sep 2024	In-person meeting	Learn from existing integrated care models in Ghent
Stakeholder Meeting – Outreach Team Coordinator	Program coordinator & research team	Nov 2024	Online meeting	Identify barriers and facilitators; provide feedback on the care model
Stakeholder Meeting – Geriatric Psychiatry Department, UPC	Research team & geriatric psychiatrists from UPC	Jan 2025	In-person meeting	Obtain feedback on the intervention model
Stakeholder Meeting –	Program coordinator &	Jan	Online meeting	Discuss implementation

Outreach Team Coordinator	research team	2025		strategies and gather further feedback on the intervention
Stakeholder Meeting – Geriatric Psychiatry Department, UPC	Research team & geriatric psychiatrists from UPC	Aug 2025	Online meeting	Align with implementation
Stakeholder Meeting – Apotheia	Rearch team & Apotheia pharmacist	Sept 2025	Online meeting	Align with implementation
